# Ecteinascidin-743: Evidence of Activity in Advanced, Pretreated Soft Tissue and Bone Sarcoma Patients

**DOI:** 10.1155/SRCM/2006/56282

**Published:** 2006-12-31

**Authors:** G. Huygh, Paul M. J. Clement, H. Dumez, P. Schöffski, H. Wildiers, J. Selleslach, J. M. Jimeno, I. De Wever, R. Sciot, L. Duck, A. T. Van Oosterom

**Affiliations:** ^1^Leuven Cancer Institute, Department of General Medical Oncology, University Hospital Gasthuisberg, Herestraat 49, 3000 Leuven, Belgium; ^2^PharmaMar SA, Avenida de los Reyes 1, Pol Ind La Mina-Norte, 28770-Colmenar Viejo, Madrid, Spain; ^3^Department of Oncology, University Hospital Saint-Luc, 1200 Brussels, Belgium

## Abstract

*Purpose*. To evaluate the activity and safety of ecteinascidin (ET-743) in pretreated patients with advanced or metastatic soft tissue and bone sarcoma. *Patients or subjects*. Eighty-nine patients received ET-743 as a 24-hour continuous infusion at a dose of 900–1500 *μ*g/m^2^ every 3 weeks. *Results*. We observed one complete remission, 5 partial remissions, one minimal response, and 16 patients with a disease stabilization of 6 months or more. The objective response rate was 6.7% and the clinical benefit rate at 3 and 6 months was 37.7% and 23.4%, respectively. Responses were noted in patients with lipo-, leiomyo-, osteo-, and myogenic sarcoma, with a median duration of 9.85 months. Toxicity mainly involved an asymptomatic elevation of transaminases and neutropenia. Estimated 1- and 2-year survival rates were 39.4% and 15.8%. Median overall survival was 8.25 months. *Discussion*. This retrospective analysis confirms that ET-743 induces objective responses and progression arrest in a clinically relevant proportion of patients.

## INTRODUCTION

Soft tissue sarcomas (STSs) represent a heterogeneous family of malignancies of mesenchymal origin, accounting for approximately 1% of all cancers worldwide each year [1]. Despite adequate treatment and control of localized disease, approximately 40% to 50% will eventually develop local recurrence or metastastic disease [2–5]. Once the tumor has progressed beyond surgical resectability, the disease is nearly always incurable, with a median survival of at best 12 months [6, 7]. With some exceptions for specific histopathologic STS subtypes such as GIST, the treatment options in this clinical setting are limited to a few cytotoxic agents like doxorubicin and ifosfamide. Although combination chemotherapy followed by metastasectomy may sometimes be curative, most patients with metastatic bone sarcoma still succumb to their disease as well. Therefore, new effective drugs in the treatment of STS and bone sarcoma are desperately needed.

Ecteinascidin 743 (ET-743; Yondelis) is a marine-derived alkaloid isolated from the Caribbean tunicate *Ecteinascidia turbinata.* It was chosen for further clinical development as an antineoplastic agent because of its unique mechanism of action, which is not yet fully understood, and its cytotoxic potency in early preclinical studies [8]. ET-743 interacts with DNA in a sequence-specific manner; it covalently binds a guanine residue in the DNA minor groove, bending the DNA helix towards the major groove [9, 10]. ET-743 inhibits gene activation via a promoter-specific mechanism [11, 12] and interacts with transcription-dependent nucleotide excision repair, inducing lethal DNA strand breaks [13]. In addition ET-743 blocks the cell cycle in the late S and G2 phases [14, 15], affects the organization and assembly of the microtubule network [16], and abrogates transcriptional activation of the *MDR 1* gene, which is involved in the development of drug resistance, while leaving constitutive gene expression relatively unaffected [11].

Preclinical studies have shown activity of ET-743 in several solid tumor cell lines and xenografts, including STSs, and demonstrated little cross-resistance with several standard chemotherapeutic agents [14, 17–19]. In the phase I clinical setting, tumor responses were observed in patients with a wide variety of malignancies, including leiomyosarcoma, liposarcoma, and osteosarcoma [20–24]. In vitro studies revealed that cytotoxicity of ET-743 was influenced by the administration schedule, with more activity with continuous exposure [14, 20, 23]. Subsequently, phase I trials showed that the administration of ET-743 in a 24-hour continuous intravenous infusion (CIV), once every 3 weeks (q3w), was better tolerated and had a higher duration of exposure compared with the 1-hour q3w and the once daily for 5 days q3w schedules [20, 24]. It also had a higher dose-intensity compared with the 72-hour CIV q3w schedule [23]. The recommended dose for phase II was 1500 *μ*g/m^2^ q3w, which was used in subsequent trials.

The demonstration of several objective responses and clinically relevant disease stabilizations among pretreated STS and bone sarcoma patients, as well as the need for effective new therapy in this clinical setting, encouraged further exploration of ET-743 in this patient population. We report here a retrospective analysis of patients with advanced and metastastic STS and bone sarcoma, treated with ET-743 in a single institution. These patients were treated either in a EORTC phase II trial or in a compassionate use program in our center. In contrast with phase II trial conducted by EORTC, we also included patients with osteosarcoma.

## PATIENTS AND METHODS

### Patient population

The present report considers two patient cohorts. Fifteen advanced, pretreated sarcoma patients were included in a phase II trial between March 1999 and September 2000. Another 74 patients, who were not eligible for the ongoing phase II trial because of histology or previous treatment or who were referred to our institution after closure of the phase II trial, were treated with ET-743 (supplied by PharmaMar, Madrid, Spain) on a named-patient basis, compassionate use program. This program as well as the phase II trial was approved by the ethics committee of the University Hospital of Leuven. Patients could be included in this program upon request of the treating physician after approval of the company.

All patients eligible for the phase II trial were required to have histologically proven, unresectable advanced, or metastastic sarcoma, excluding the following histological subtypes: chondrosarcoma, neuroblastoma, osteosarcoma, Ewing's sarcoma, malignant mesothelioma, and embryonal rhabdomyosarcoma. Patients with gastrointestinal stromacell tumors (GIST) received ET-743 as first-line treatment, as Imatinib was not available at that time. The other patients were pretreated with one line of previous single agent chemotherapy, which had to be discontinued for more than 4 weeks (adjuvant chemotherapy was not considered as first line, unless the patient progressed within six months following adjuvant chemotherapy).

All patients were required to have at least one measurable lesion located in a nonirradiated area, with evidence of progression within 6 weeks prior to treatment (osseous lesions, pleural effusions, and ascites were not considered measurable). Other eligibility criteria included the following: age ≥18 years, ECOG PS of <2, adequate bone marrow reserve (neutrophil count ≥ 2 × 10*9/ l and platelet count ≥ 100 × 10*9/ l), normal renal and hepatic function (serum creatinine ≤120 *μ*mol/l or calculated creatinine clearance (Cockroft formula) ≥60 ml/min, AST and ALT < 1.5 × ULN in case of no liver metastasis or < 2.5 × ULN in case of liver metastasis, alkaline phosphatase ≤ULN, bilirubine ≤ULN, albumine ≥25 g/l), no other severe medical illness, no central nervous system (CNS) metastases, and no prior or concurrent second primary malignant tumors (except adequately treated in situ carcinoma of the cervix or basal cell carcinoma). Fertile males and females were to use medically approved contraception, pregnant or lactating women were excluded. Patients were excluded if regular follow-up attendance was impractical. All patients were required to have an indwelling central venous access device (eg, port-a-cath) in place for drug administration. A signed written informed consent was obtained from each patient before accrual.

The same inclusion criteria applied to patients entered onto the compassionate use program, with the exception of the following conditions, which were accepted in the compassionate use program and not in the phase II trial: age <18 (with parental authorization), ECOG PS 2, decreased blood cell count, decreased renal or hepatic function, CNS involvement, and pretreatment with more than 1 single-agent or combination therapy. All subtypes of sarcoma were allowed. For patients with angiosarcoma, previous treatment with taxanes was recommended and for patients with GIST pretreatment with imatinib.

We further categorized our population in terms of the anthracycline resistance (defined as progression occurring while under the anthracycline-containing treatment, within 3 months of completing palliative treatment or within 6 months of completing adjuvant treatment), and bulky disease (defined as the existence of at least one tumor mass with a diameter of at least 10 cm). The phase II and compassionate use patients were pooled for the purpose of this retrospective analysis.

### Treatment plan

The primary endpoint of our analysis was to determine the response rate and duration of response to ET-743 in advanced STS and bone sarcoma patients. The secondary endpoint was to further characterize the toxicity profile of ET-743 in this patient population. We also report on the clinically relevant time-related parameters such as progression-free survival (PFS) and overall survival (OS), for the entire population and the STS subgroup.

Prestudy assessments were to be performed within 14 days before initiating therapy, including the following: medical history, physical examination, performance status, complete differential blood cell counts and blood chemistry (urea, creatinine, sodium, potassium, calcium, glucose, AST, ALT, alkaline phosphatase, bilirubin, LDH, and albumine), urinanalysis (dip stick), chest X-ray, and radiologic evaluation of all measurable or assessable sites. Complete differential blood cell counts and liver function tests were repeated weekly; AST and ALT were also measured on day 3 of each cycle.

Physical examination, performance status, complete differential blood cell counts, blood chemistry, and urinanalysis, were performed before each cycle of therapy. Toxicity was evaluated in each cycle and graded according to the National Cancer Institute—Common Toxicity Criteria (NCI-CTC), version 3.0. Tumor response was evaluated every 2 cycles until disease progression according to the WHO criteria in the phase II trial and RECIST criteria in the compassionate use program. Patients in the phase II trial who achieved complete or partial response were reevaluated 4 weeks later to confirm the initial observation of response. Responses were reviewed by independent experts in the phase II trial, not in the compassionate use program.

ET-743 was supplied as a sterile lyophilized product in a clear vial, containing 250 *μ*g of ET-743 with 250 mg of mannitol, 34 mg of monopotassium phosphate, and phosphoric acid to ajust pH until 4.00. Each vial was reconstituted with 5 ml of sterile water for injection. The reconstitution solution was further diluted in the desired amount of normal saline and administered intravenously via a central venous catheter, without the use of an inline filter.

In the phase II trial, ET-743 was administered at a dose of 1500 *μ*g/m^2^ as a 24-hour CIV, repeated every 3 weeks. In the compassionate use program, the starting dose of ET-743 was reduced (900–1350 *μ*g/m^2^) in the following cases: insufficient haematological reserve, alkaline phosphatase elevation, bilirubin elevation, creatinine elevation, performance status 2, and heavily pretreated patients. If the reduced dose was well tolerated, the following cycle could be given at a higher dose level. Dexamethasone 4 mg was given orally every 12 hours from the day before the administration until day 2. Before the treatment, 20 mg of dexamethasone and ondansetron 8 mg were given intravenously. Other antiemetic agents such as lorazepam or metoclopramide could be administered approximately 15 minutes to two hours before the ET-743 infusion.

The treatment was administered every 3 weeks unless there was insufficient haematological recovery (ANC < 2 × 10*9/ l, platelets < 100×10*9/ l), unless creatinine, transaminases, and bilirubin were not yet returned to normal or baseline values, or unless grade 4 nonhematological toxicity was not yet recovered, in which case treatment was interrupted for up to 2 weeks until recovery. Treatment delays for more than 2 weeks led to withdrawal from the study. In the compassionate use program, a longer delay was allowed. Dose adjustments were based on the most severe toxicity noted in the previous cycle. The dose of ET-743 was reduced to 1200 *μ*g/m^2^ in the phase II trial or to a lower dose level in the compassionate use program in the following cases: febrile neutropenia, grade 4 neutropenia lasting more than 5 days, grade 4 thrombocytopenia, any nonhematological grade 3 to 4 toxicity (except grade 3 to 4 elevated AST/ALT), and ≥grade 1 increase of bilirubin or alkaline phosphatase. If during a subsequent cycle there was a further episode of toxicity requiring a new dose reduction, the dose was reduced to 1000 *μ*g/m^2^ in study patients or a lower dose level in the compassionate use patients. Prophylactic use of cytokines (G-CSF) was allowed only in case of febrile neutropenia occurring in the prior cycle of treatment and if the dose had already been reduced. Treatment with ET-743 was continued until disease progression, patients refusal or excessive toxicity precluding further therapy, according to the responsible physician.

### Statistical methods

Descriptive statistics were used to characterize response and toxicity rates. The response rate was estimated as the proportion of patients who achieved a complete or partial response among all patients who received at least one cycle of ET-743. Two-stage conditional exact binomial 95% confidence intervals (CI) were used to describe the distribution of the response rate. *χ* ^2^ was used for comparison of toxicities between the phase II trial and the CU group.

Overall survival (OS), time to disease progression (TTP), and duration of response were estimated according to the Kaplan-Meier product-limit method. TTP was defined as the time from initiation of therapy to the first documentation of disease progression. OS was defined as the time between the first study treatment and death. Duration of response was defined as the time between the first documentation of objective response and documentation of disease progression. Duration of stable disease was measured from the start of the treatment until criteria for progression were met.

## RESULTS

### Patient characteristics

Between March 1999 and September 2000, 15 patients (7 women, 8 men) were treated in the phase II trial. Seventy-four patients (33 women, 41 men) were treated in the compassionate use program between September 1999 and September 2004. At the time of analysis, 9 patients were still on treatment with ET-743. Patient characteristics are summarized in Table 1. The median age was 51 years (range, 16 to 76 years). The majority of patients was in good general condition (84 patients ECOG PS 0 or 1; 5 patients ECOG PS 2). The most common tumor types were leiomyosarcoma (29 patients; 33%; 4 of which were of uterine origin), liposarcoma (16 patients; 18%), and osteosarcoma (14 patients; 16%) (Table 1). All patients had metastastic or locally advanced disease with a median of 3 involved sites (range, 1 to 7 sites), the most common sites being lung or pleura (71% of patients), soft tissue (36% of patients, including primary site of disease), lymph nodes (31% of patients), liver (28% of patients), and bone (22% of patients, including primary site of disease). Twenty-nine % of patients had bulky disease. The median time between sarcoma diagnosis and initiation of treatment with ET-743 was 24.0 months (range, 0.6 to 300 months).

The patients had received a median of 2 prior chemotherapy regimens (range, 0 to 6 regimens; Table 2). Three patients received ET-743 as first-line treatment: one patient with a leiomyosarcoma, one with a liposarcoma, and one with GIST. Most patients (93%) had been previously treated with anthracyclines, of which 55% were clinically resistant and 77% were subsequently treated with ifosfamide. Seventy-four % of the patient population had received prior ifosfamide therapy and 42% prior radiotherapy (Table 2).

### Drug delivery

A total of 430 cycles of ET-743 were administered at the time of this analysis, with a median number of 2 cycles for an individual patient, ranging from 1 to 31 cycles. Thirteen cycles (3%) were dose reduced for the following reasons: self-limited transaminitis (8 cycles), neutropenia (2 cycles), thrombocytopenia (1 cycle), and combination of thrombocytopenia and neutropenia (2 cycles). The administration of 91 cycles (21%) was delayed because of neutropenia (27 cycles); thrombocytopenia (10 cycles); combination of thrombocytopenia and neutropenia (2 cycles); self-limited transaminitis (15 cycles); combination of transaminitis and neutropenia (5 cycles); combination of transaminitis, neutropenia, and thrombocytopenia (1 cycle); infection (2 cycles); and nonmedical reasons, such as holiday or patients request (29 cycles).

At the time of this analysis, 80 patients have discontinued treatment. The reasons for discontinuation included disease progression in 63 patients (70%), elective surgery in 5 patients (6%), patient withdrawal in 6 patients (7%), and toxicity in 6 patients (7%): one long lasting thrombocytopenia grade 2, 1 methicilline resistant staphylococcus aureus (MRSA) sepsis, 1 pancytopenia complicated with a fatal gastrointestinal hemorrhage, 1 septic shock, and 2 patients with asthenia and anorexia grade 3. Twenty-four (27%) of patients received 6 cycles or more of ET-743.

### Efficacy

The objective response rate is shown in Table 3. Tumor response could not be evaluated in 7 patients due to early discontinuation of the treatment for the following reasons: toxic death (2), long lasting hematotoxicity (2), and withdrawal of consent for further treatment (3).

Among the 89 patients who received at least one dose of ET-743, 6 objective responses were seen including one complete remission and 5 partial responses (objective response rate 6.7%; 95% CI, 1.4% to 12.1%). The complete remission was seen in a patient with a myxoid liposarcoma after 6 cycles, lasting for 8.7 months at the moment of the analysis, and the patient is still continuing treatment. The partial responses occurred in two patients with a leiomyosarcoma, of which one was of uterine origine, in one patient with a myxoid liposarcoma, one patient with a HG osteosarcoma, and one patient with a myogenic sarcoma. One patient (with a myxoid liposarcoma) received ET-743 as first-line treatment, all other responding patients had received prior anthracyclines, of whom one was clinically resistant. Four of the responding pretreated patients also received prior treatment with ifosfamide. Two patients who experienced partial remission exhibited liver metastases and one patient bulky disease. Median duration of response was 9.85 months (range, 2 to 43.5 months). One minor response (40% tumor reduction) was observed in a patient with a synovial sarcoma, lasting for 5.8 months. Responses were seen in lung, liver, retroperitoneal, and abdominal localizations, as well as in lymph nodes and soft tissue. The characteristics of responding patients are summarized in Table 4. Furthermore, 16 patients experienced disease stabilization for 6 months or more (median 8.75 months; range, 6.8 to 45 months), of which one patient with an alveolar soft part sarcoma.

One patient with osteosarcoma and lung metastasis went off study 3 months after achieving a partial remission, having received 8 cycles of ET-743, for an attempt of salvage surgery, which was not successful. She eventually progressed one year after treatment discontinuation and was retreated with ET-743, which was discontinued after another 9 cycles because of only stabilization of disease and fatigue. Disease progression was noted 7 months after restarting treatment with ET-743, 2 months after discontinuation of ET-743. Two patients (one alveolar soft part sarcoma and one leiomyosarcoma) continued treatment at disease progression, respectively, after 18 and 13 cycles, leading to a marked slowering of the growth speed, which was documented by comparing CT scans under treatment with ET-743 with those under previous treatments. Both patients are still under treatment with ET-743 at the moment of the analysis and have received 31 and 27 cycles of ET-743, respectively.

For 9 patients with liver or lung metastasis, intraabdominal, or retroperitoneal localization of disease, the therapeutic impact of ET-743 permitted salvage surgery attempts. Seven patients were rendered macroscopically tumor-free, of which 2 underwent surgery a few days before the end of the analysis, the other 5 remained progression-free for 40, 36, 6, 6, and 1 months, respectively. One patient with a myogenic sarcoma achieved a partial remission after 4 cycles of treatment. He underwent a complete resection after 8 cycles, followed by radiotherapy and 4 cycles of ET-743 in an adjuvant setting, after which disease recurrence developed.

**Figure 1:**
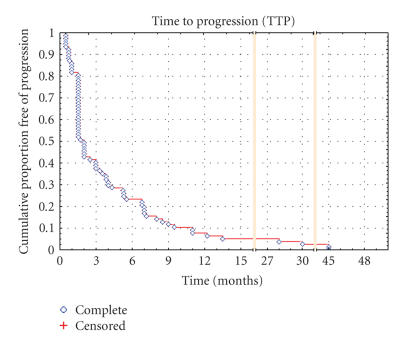
Kaplan-Meier curve of time to progression for the entire population.

**Figure 2:**
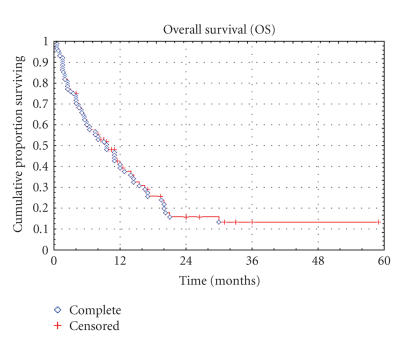
Kaplan-Meier curve of overall survival for the entire population.

**Figure 3:**
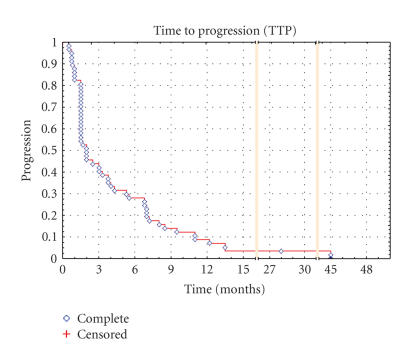
Kaplan-Meier Curve of time to progression for the STS subgroup.

**Figure 4:**
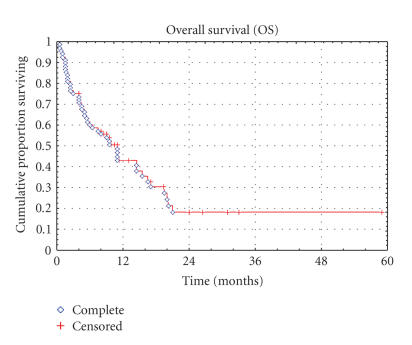
Kaplan-Meier Curve of overall survival for the STS subgroup.

Time-related parameters were updated until September 30, 2004. With a median follow-up of 8.25 months (range, 0.5 to 59 months), 63 patients have progressed and 66 patients died. Median TTP and OS in all patients were 2.0 months (range, 0.5 to 45 months) and 8.2 months (range, 0.5 to 59 months), respectively (Figures 1 and 2). 37.7% and 23.4% of the patients were progression-free at 3 and 6 months, and the OS rate at 1 and 2 years was 39.4% and 15.8%, respectively. For the STS subgroup, the median TTP and OS were 2.0 months (range, 0.5 to 45 months) and 8.75 months (range, 0.5 to 59 months), respectively, the PFS at 3 and 6 months were 43.9% and 28.1%, with a 1- and 2-year survival rate of 43.0% and 18.2%. The TTP and OS in the STS population are shown in Kaplan-Meier plots in Figures 3 and 4, respectively.

### Safety

All patients were assessed for safety. Hematologic and nonhematologic toxicities are listed in Tables 5 and 6. The predominant hematologic toxicity was neutropenia, reaching grade 3 to 4 in 26% and 12% of the patients, respectively. Only 7 patients developed a febrile neutropenia episode with need for hospitalization and intravenous antibiotic administration, with need for G-CSF administration in one patient. One patient died as a result of septic shock. Anemia and thrombocytopenia reached grade 3 to 4 in 7% and 18% of patients, respectively. One patient experienced a gastrointestinal hemorrhage due to prolonged grade 4 thrombocytopenia. In 3 patients grade 2 thrombocytopenia lasted for more than one month, leading to discontinuation of therapy in one of them. Transfusion of packed red blood cells and platelets was required in 9 and 5 patients, respectively, but was possibly not systematically reported for the compassionate use group.

An acute self-limiting transaminitis was frequently observed, with 39% and 3% of patients developing grade 3 and 4 elevation of ALT and AST, respectively. AST and ALT peaked 3 to 5 days after completing the ET-743 infusion, and resolved in almost all cases before the start of the next cycle (15 cycles delayed because of transaminitis). In one patient it was associated with asthenia and emesis grade 3, mandating hospitalization and eventually discontinuation of therapy. An elevation of bilirubin and/or alkaline phosphatase was less frequent (resp, in 6% and 7% of patients, in only one patient, bilirubin and alkaline phosphatase were both elevated), resulting in drug delay in these patients. Renal failure occurred in one patient, secondary to a gastrointestinal bleeding.

Other toxicities experienced during treatment are listed in Table 6. Fatigue was a common side effect, NCI-CTC grade 2 and 3, respectively, in 35% and 13% of the patients. Nausea and vomiting were mild to moderate (grade 2 and 3 in 24% and 4% of the patients, resp) with the routine use of antiemetics. Other toxicities were rare, such as alopecia (grade 1 and 2 both in one patient), mucositis (grade 1, 2, and 3, resp, in 2, 3, and 1 patients) and diarrhea (grade 2 and 3 in, resp, 4 and 1 patients). Toxicities, hematologic as well as nonhematologic, were less frequent in the patients treated in compassionate use, reaching significance for neutropenia, anemia, transaminitis, anorexia, nausea, and vomiting. All biochemical toxicities were reassessed by an independent expert. For nausea, vomiting, and anorexia, a possible reporting bias must be considered. The lower incidence of toxicity may also be explained in part by the lower mean dose per cycle in the population that was treated per compassionate use protocol.

Ten treatment-related serious adverse events occurred, leading to death in two patients (both treated in the phase II trial), consisting of 7 episodes of febrile neutropenia (complicated with a brain abcess, MRSA sepsis, and a fatal septic shock), 2 gastrointestinal hemorrhages due to grade 3 and 4 thrombocytopenia, leading to renal failure and death in one patient, and emesis and asthenia grade 3 in combination with transaminitis grade 3 in one patient.

## DISCUSSION

The treatment of unresectable advanced or metastatic soft tissue sarcoma (STSs) remains a challenge for medical oncologists. Doxorubicin and ifosfamide represent the two most active conventional agents in the treatment of STSs, both showing response rates of 20–30% in non-pretreated patients [2–4, 25, 26]. Doxorubicin remains the first-line treatment of choice, as a single agent or in combination with ifosfamide. However, its use is precluded by the risk of cardiotoxicity. Ifosfamide is therefore the treatment of choice for patients who have received the maximum-tolerated cumulative dose of anthracyclines or after treatment failure with anthracyclines. Response rates of ifosfamide in second-line treatment unfortunately are much lower, varying from 6% to 16% [27, 28]. Dose intensification and combination therapy may increase the response rate, with respective response rates reaching 39-40% [29–31] and 45% [32] in phase II trials, but at the expense of a substantial increase of toxicity without impact on survival. In the palliative setting of STS, these more aggressive approaches can therefore not be recommended [27, 33].

Currently, no reliable therapeutic options exist after failure of treatment with anthracyclines and ifosfamide. All newly available cytotoxic agents tested in pretreated and non-pretreated patients have shown disappointing results, with the exception of specific tumor types such as GIST, for which targeted therapies are available. This report demonstrates that a new marine-derived cytotoxic agent, ET-743, is active in a subset of previously treated patients with STS and bone sarcoma, showing a response rate of 6.7%, with acceptable toxicity. Our results are in concordance with preliminary results of phase I and II trials that demonstrated response rates ranging from 4% to 13% [5, 21, 34–36]. The median overall survival of 8.25 months is also comparable with results from other phase II trials [5, 21, 34]. Responses were seen in patients with bulky disease, anthracycline resistance, and liver metastases, known to be an adverse prognostic factor for response and survival [6, 7], and were observed in multiple disease sites, including lung, liver, and soft tissue.

Among the 89 patients, one complete remission and 5 partial responses (lasting for a median of 9.85 months) were achieved. One further patient experienced a clinically relevant minor response (40% tumor reduction), lasting for 5.8 months. Although numerically, the response rate is low (6.7%), responses are durable, and 23.4% of patients with proven disease progression at initiation of treatment with ET-743 were progression-free at 6 months. As suggested earlier, clinical benefit (CR+PR+MR+SD) could be a clinically more relevant indicator of activity in the palliative setting of advanced STS and bone sarcoma [36]. Indeed, the EORTC Soft Tissue and Bone Sarcoma Group (STBSG) has published data that indicate that the variables predicting survival do not necessarily correlate with the ones that predict objective response to therapy [6], consistent with the finding in a retrospective analysis of 1154 patients that progression-free survival at 3 and 6 months correlates with overall survival, even in the absence of objective response [37].

The clinical benefit of ET-743 in our patient cohort, although not mentioned as a main endpoint of this analysis at the time of its conception, was 37.7% at 3 months and 23.4% at 6 months for the entire population and 43.9% and 28.1%, respectively, for the STS subgroup. These results are consistent with the results of a phase II trial conducted by Yovine et al in 36 patients that demonstrated clinical benefit of 38.8% and 24.1% at 3 and 6 months, respectively [5]. Median TTP in the entire population and the STS subgroup were 2.0 months (range, 0.5 to 45 months) (Figure 1).

Adult soft tissue sarcomas, often with osteosarcoma, have generally been grouped together in clinical trials because of the rarity of the distinct histologic subtypes. However, important differences in response to therapy exist between histologic subsets of STSs. In a multivariate analysis of prognostic factors performed by the EORTC STBSG, liposarcoma histology was found to be an independent prognostic factor of response to chemotherapy [6]. It should be mentioned that 2 major responses observed in our patient cohort, 1 CR and 1 PR, occurred in patients with liposarcoma, both of the myxoid subtype. This represents a response rate of 12.5% for the subset of 16 patients with a liposarcoma included in this analysis. If we consider disease control, a clinical benefit of 62.5% at 3 months and 31.3% at 6 months was calculated for this histologic subtype. Garcia-Carbonero et al postulated that the particular sensitivity of the myxoid liposarcomas may be the result of a molecular mechanism related to the DNA minor groove-binding activity of ET-743 and its interaction with an aberrant transcriptional regulator, generated from a translocation between chromosomes 12 and 16, present in this histologic subtype [33]. Worth mentioning is that the patient with liposarcoma who experienced a partial response was treated upfront with ET-743. It was already suggested by a study presented at ASCO in 2000 that ET-743 in first-line treatment may have higher response rates, with a reported response rate of 18% in that trial [38].

Interestingly, responses were not only seen in patients with liposarcoma, but also in leiomyosarcomas, which have been reported to be relatively chemotherapy-resistant, particularly those of visceral origin with liver metastasis [6]. Two patients with a leiomyosarcoma, of which one was of uterine origin and the other displayed liver metastasis, developed a partial response. A third patient with partial remission had a myogenic sarcoma, possibly a leiomyosarcoma, or a myofibroblastic sarcoma. One minor response and 4 long lasting stabilizations were observed in patients with synovial sarcoma, leading to a clinical benefit at 3 and 6 months of 50% and 25%, respectively.

Our observations of best response in liposarcoma, leiomyosarcoma, and synovial sarcoma are consistent with preliminary reports from phase II trials using ET-743 in this patient population [5, 21, 33, 34]. Most clinical trials excluded patients with osteosarcoma, explaining the very limited experience with ET-743 in this patient population. For several years, new chemotherapeutic single agents were tested in this tumor type, with disappointing results [19]. Laverdiere et al did demonstrate 3 minor responses among 24 patients with osteosarcoma in a nonrandomized phase II trial with ET-743 [39]. Ruiz-Casado and Delaloge reported partial responses in 2 of 17 patients and 2 of 3 patients with osteosarcoma [21, 34]. In our patient population, one patient with osteosarcoma and lung metastasis achieved a partial remission after 4 cycles of ET-743. After 8 cycles of ET-743, she was referred to a surgeon for salvage surgery, which was not successful. She eventually progressed 1 year after treatment discontinuation and experienced disease stabilization for another 7 months under retreatment with ET-743, after which she decided to discontinue treatment because of fatigue. These promising results certainly merit further attention.

Also surprising was the long lasting (13.5 months) disease stabilization in one patient with an adult soft part sarcoma, a histologic subtype reported to be resistant to chemotherapy [6], but which can be stable for a long time without treatment. Even after disease progression, the continuation of ET-743, which was approved by the company, clearly slowed down the growth of the tumor, which was assessed by comparing CT scans under treatment with ET-743 with those under previous treatments.

Treatment with ET-743 was overall well tolerated and toxicities encountered in our patient population were mostly manageable, as was documented by the fact that only 6 patients discontinued treatment because of treatment-related toxicity. Neutropenia was the most frequently occurring serious side effect (grade 3 and 4 in 26% and 12% of patients, resp), complicated by febrile neutropenia in 7 patients. Thrombocytopenia was mild, leading to drug delay in 13 cycles. A transient transaminitis, with 39% and 3% of patients developing grade 3 and 4 elevation of ALT and AST, respectively, led to dose reduction in 8 cycles and treatment delay in 21 cycles, but never necessitated treatment discontinuation. Other toxicities were mild and usually transient. The low rate of alopecia (only in two patients) is of note. We observed higher toxicity in the group of patients treated in the phase II trial, in which two toxic deaths occurred: one gastrointestinal hemorrhage due to thrombocytopenia grade 4 and one neutropenic septic shock. The better tolerability in the compassionate use group can be explained by starting dose adjustments that were made according to pretreatment, performance status, haematology, and liver and renal function, which were implemented after the identification of biochemical parameters predictive for the occurrence of severe toxicities [40]. For the non-biochemical side effects, however, differences between the 2 groups may be partly due to reporting bias. There was an equal tolerability between patients who were highly pretreated and patients who received ET-743 as first-, second-, or third-line treatment. We confirmed hereby the observation of Yovine that even heavily pretreated patients tolerate this regimen well when guidelines for patient selection and dose adjustments are respected [5].

In conclusion, this report shows that ET-743, in a 24-hour CIV regimen, induces long lasting objective remissions and tumour control in a clinically relevant proportion of advanced sarcoma, resistant to or relapsed under conventional therapies. Further evaluation of the activity of ET-743 in sarcomas is therefore warranted, not only after failure of conventional chemotherapy, but also in earlier stages of the disease and in combination regimens. The activity of ET-743 is not limited to STS, but is also seen in osteosarcoma. Further research on predictive factors for response to ET-743 will be useful to better select which patients to treat with this new drug.

## Figures and Tables

**Table 1:** Patient characteristics at baseline. PS: performance status; ECOG: eastern cooperative oncology group; HG: high grade.

	Group 1^(a)^	Group 2^(b)^
	(*n* = 15)	(*n* = 74)	All (*n* = 89)
	Number of patients (%)	

Age, years
< 40	3 (20)	20 (27)	23 (26)
40–60	4 (27)	38 (51)	42 (47)
> 60	8 (53)	18 (22)	24 (27)
Median	61	51	51
Range	21–76	16–74	16–76

Sex
Female	7 (47)	33 (45)	40 (45)
Male	8 (53)	41 (55)	49 (55)

PS (ECOG)
0	4 (27)	28 (38)	32 (36)
1	11 (73)	41 (55)	52 (58)
2	0 (0)	5 (7)	5 (6)

Tumor histology
Leiomyosarcoma	5 (33)	24 (32)	29 (33)
Nonuterine	5 (33)	20 (27)	25 (28)
Uterine	0 (0)	4 (5)	4 (5)
Liposarcoma	1 (7)	15 (20)	16 (18)
Osteosarcoma	0 (0)	14 (19)	14 (16)
Synovial sarcoma	4 (27)	3 (4)	7 (8)
HG sarcoma	3 (20)	3 (4)	6 (7)
Other	2 (13)	15 (20)	19 (21)

Grade
High	11 (73,3)	36 (48,6)	47 (52,8)
Intermediate	1 (6,7)	5 (6,7)	6 (6,7)
Low	0 (0)	3 (4,1)	3 (3,3)
Unknown	3 (20)	30 (40,5)	33 (37,1)

Bulky disease^(c)^
Yes	3 (20)	23 (31)	26 (29)
No	12 (80)	51 (69)	63 (71)

Number of sites involved
Median	2	3	3
Range	1–4	1–7	1–7

Disease localization
Lung or pleura	11 (73)	52 (70)	63 (71)
Soft tissue	5 (33)	27 (36)	32 (36)
Lymph node	7 (47)	21 (28)	28 (31)
Liver	3 (20)	22 (30)	25 (28)
Bone	4 (27)	16 (22)	20 (22)

Time since initial
diagnosis (months)
Median	14	24	24
Range	0.6–90	4–300	0.6–300
< 12	6 (40)	14 (19)	20 (22)
12–36	5 (33)	22 (30)	27 (30)
> 36	4 (27)	28 (38)	32 (36)

^(a)^Group 1: patients treated in the phase II trial.

^(b)^Group 2: patients treated in a compassionate use program.

^(c)^Existence of at least one tumor mass with a diameter of at least 10 cm.

**Table 2:** Prior treatment.

	Group 1	Group 2
Type of treatment	(*n* = 15)	(*n* = 74)	All (*n* = 89)
	Number of patients (%)	

Number of prior chemotherapy regimens
0	1 (7)	2 (3)	3 (3)
1	10 (67)	25 (34)	35 (39)
2	4 (27)	29 (39)	33 (37)
≥ 3	0 (0)	18 (24)	18 (20)
Median	1	2	2
Range	0–2	0–6	0–6

Prior chemotherapy
Anthracyclines	13 (87)	70 (95)	83 (93)
Ifosfamide	8 (53)	59 (80)	67 (75)

Anthracycline clinical resistance^(a)^
Resistant	9 (60)	37 (50)	46 (52)
Sensitive	4 (27)	33 (45)	37 (41)
Never exposed	2 (13)	4 (5)	6 (7)

Prior radiotherapy
Yes	5 (33)	32 (43)	37 (42)
No	10 (67)	42 (57)	52 (58)

^(a)^Anthracycline resistance: progression occurring while under anthracycline-containing treatment, within 3 months of completing palliative treatment or within 6 months of completing adjuvant treatment with anthracyclines.

**Table 3:** Best response. CR: complete remission; PR: partial remission; MR: minimal response; SD: stable disease; PD: progressive disease; NE: not evaluated; ORR: overall response rate.

	Group 1	Group 2
Response	(*n* = 15)	(*n* = 74)	All (*n* = 89)
	Number of patients (%)	

CR	0 (0)	1 (1)	1 (1)
PR	1 (7)	4 (5)	5 (6)
MR	1 (7)	0 (0)	1 (1)
SD ≥ 6 months	4 (27)	12 (16)	16 (18)
SD ≥ 2 and < 6 months	1 (7)	15 (20)	16 (18)
PD	5 (33)	38 (51)	43 (48)
NE	3 (20)	4 (5)	7 (8)
ORR	6.7%	6.7%	6.7%

Clinical benefit, CR + PR + MR + SD
≥ 3 months	7 (53)	27 (36)	34 (38)
≥ 6 months	5 (33)	16 (23)	21 (23)

**Table 4:** Characteristics of responding patients. CR: complete remission; PR: partial remission; MR: minor response; SD: stable disease; M: male; F: female; LMS: leiomyosarcoma; S: sarcoma; GIST: gastrointestinal stromacell sarcoma; ASPS: alveolar soft part sarcoma; ST: soft tissue; Abd: abdominal; L: lung/pleura; Li: liver; Ret: retroperitoneal; P: peritoneal; LN: lymph nodes; Th: thyroid; K: kidney; B: bone; br: brain.

Best response	Patients number (%)	Sex	Age (years)	Histology	Disease sites	Number of previous lines	Anthracycline resistance^(a)^	Bulky^(b)^	Response duration (months)

CR	1 (1.1)	M	57	Liposarcoma	ST, Abd	1	N	N	8.7^+^

PR	5 (5.6)	F	71	LMS	L	1	Y	N	43.5
		F	61	Osteosarcoma	L	2	N	N	15
		M	46	Myogenic	Li, LN, ST	2	N	N	11
		F	38	LMS uterus	Li, L, ST, P	1	Y	N	7.3
		M	41	Liposarcoma	Ret, P	0	—	Y	2.7^+^

MR	1 (1.1)	F	76	Synovial S	L	1	N	N	5.8

SD ≥ 6 months	16 (17.9)	F	37	GIST	Abd	0	—	N	43.5
		F	56	LMS	LN, ST, L, B	2	N	Y	19.3^+^
		M	35	LMS	L	2	N	N	12.5
		M	61	LMS	Li, B	1	Y	N	11
		M	76	LMS	L, Li, ST, K	1	N	Y	7
		F	42	LMS	L, Li, ST	1	N	N	7
		F	60	LMS uterus	ST	2	Y	N	6.8
		F	31	Liposarcoma	Th, Abd	2	N	Y	12.2
		F	58	Liposarcoma	Abd	1	Y	Y	8.5^+^
		M	58	Liposarcoma	Abd	2	N	Y	8
		M	53	Liposarcoma	Retr, K	3	N	Y	8^+^
		F	40	Synovial S	LN, ST, Li, P	1	N	N	7
		M	21	Synovial S	L, LN	1	Y	N	6.8
		M	46	Spindle cell S	L, B, skin	2	Y	N	17^+^
		M	29	ASPS	L	1	N	N	13.5
		F	3	Osteosarcoma	B, ST, br	2	Y	N	9

^(a)^Anthracycline resistance: progression occurring while under anthracycline-containing treatment, within 3 months of completing palliative treatment or within 6 months of completing adjuvant treatment with anthracyclines.

^(b)^Bulky disease: existence of at least one tumor mass with a diameter of at least 10 cm.

^+^: Patients still undergoing treatment with ET-743, disease progression not yet reached.

**Table 5:** Hematologic toxicities (NCI-CTC grade) per cycle and per patient. NCI-CTC: National Cancer Institute Common Toxicity Criteria; Gr: grade; FN: febrile neutropenia; NS: not significant.

		Neutropenia		Thrombocytopenia		FN	Anemia	
	Total	Gr 3	Gr 4	Gr 3	Gr 4		Gr 2	Gr 3-4
		Number of patients (%)						

Per cycle
Phase II	59	24 (40.7)	9 (15.3)	3 (5.1)	2 (3.4)	2 (3.4)	15 (25.4)	1 (1.7)
CU	372	20 (5.4)	8 (2.2)	10 (2.7)	3 (0.8)	5 (1.3)	33 (8.8)	5 (1.3)
Total	431	54 (12.5)	17 (3.9)	13 (3.0)	5 (1.2)	7 (1.6)	48 (11.1)	6 (1.4)
*χ* ^2^, *P* value		< .0001	< .0001	NS	.08	NS	.0002	NS

Per patient
Phase II	15	10 (66.7)	5 (33.3)	3 (20)	2 (13.3)	2 (13.3)	6 (40)	1 (6.7)
CU	74	11 (14.9)	6 (8.1)	8 (10.8)	3 (4.1)	5 (6.7)	18 (24.3)	5 (6.7)
Total	89	21 (23.6)	11 (12.3)	11 (12.3)	5 (5.6)	7 (7.9)	24 (26.9)	6 (6.7)
*χ* ^2^, *P* value		< .0001	< .0001	NS	NS	NS	NS	NS

**Table 6:** Nonhematologic toxicities (NCI-CTC grade) per cycle and per patient. NCI-CTC, National Cancer Institute Common Toxicity Criteria; Gr, grade; NS, not significant.

		Transaminitis		Bilirubin	Nausea/vomiting		Anorexia		Asthenia	
	
	Total	Gr 3	Gr 4	Gr 2-3	Gr 2	Gr 3	Gr 2	Gr 3	Gr 2	Gr 3
		Number of patients (%)								

Per cycle
Phase II	59	19 (32.2)	0 (0)	0 (0)	15 (25.4)	2 (3.4)	8 (13.6)	5 (8.5)	6 (10.2)	4 (6.8)
CU	372	59 (15.9)	5 (1,4)	5 (1.4)	23 (6.2)	2 (0.5)	21 (5.6)	3 (0.8)	61 (16.4)	10 (2.7)
Total	431	78 (18.1)	5 (1.2)	5 (1.2)	38 (8.8)	4 (0.9)	29 (6.7)	8 (1.8)	67 (15.5)	14 (3.2)
*χ* ^2^, *P* value		.027	NS	NS	< .0001	.051	NS	.0001	NS	NS

Per patient
Phase II	15	10 (66.7)	0 (0)	0 (0)	7 (46.7)	2 (13.3)	5 (33.3)	5 (33.3)	5 (33.3)	4 (26.7)
CU	74	25 (33.8)	5 (6.7)	5 (6,7)	14 (18.9)	2 (2.7)	14 (18.9)	3 (4.1)	29 (39.2)	8 (10.8)
Total	89	35 (39.3)	5 (5.6)	5 (5.6)	21 (23.6)	4 (4.5)	19 (21.3)	8 (8.9)	34 (38.2)	12 (13.5)
*χ* ^2^, *P* value		.02	NS	NS	.02	.07	NS	.0006	NS	NS
